# Pseudo-Chancre

**Published:** 1895-11

**Authors:** William S. Gottheil


### Current Medical Literature.

### Clinics.

PSEUDO-CHANCRE.—By William S. Gottheil, M. D. Read before the Section of Dermatology and Syphiligraphy at the meeting of the American Medical Association, Baltimore, May 8th, 1895. (*New York Medical Journal*, September 28th, 1895.)

Reinfection syphilitica does occur, but the recorded cases that are entirely trustworthy are very few indeed. Analysis shows that most of the alleged cases are open to grave doubt, and that some of them are manifestly errors of diagnosis.

The following lesions may simulate chancre:

a. Artificial indurations caused by irritants applied to simple lesions.

b. Nodular lymphangites, as occur in gonorrhœa.

c. Scabies, where penile lesions are the rule.

d. Secondary indurations at the site of the initial lesion (Fournier's pseudo-chancre).

e. Secondary syphilitic papules or tubercles situated upon the genitals.

f. Ulcerative gummata of the genitals.

g. Epitheliomata of the genitals.

Two such cases have recently come under the author's observation. In the first one a non-specific sore was irritated with cauterisants until it exactly resembled a sclerosis, and was so diagnosticated by competent authorities. Nevertheless it healed up under local treatment alone; and until now, two years after date, no secondary symptoms have appeared.

The other case was one of gumma of the penis in a subject in the tertiary stage of syphilis. The lesion resembled an initial one very closely, and was at first regarded as such; but a close examination showed the presence of evidences of past specific disease, and this was confirmed by the history. The entire lesion melted away under the iodide of potassium.

Conclusions:

1. There is no characteristic sign, and no characteristic combination of signs, that enables us to diagnosticate a chancre from the lesion alone.

2. Only the advent of other syphilitic symptoms enables us to form an opinion as to the presence of systemic infection.

3. Almost all the alleged cases of syphilitic re-infection are of doubtful validity; and most of them are pseudo-chancres belonging to one or other of the above varieties.

